# Treatment failure and the threshold of disease extinction

**DOI:** 10.1016/j.idm.2024.12.007

**Published:** 2024-12-17

**Authors:** Pichaya Voottipruex, Nichaphat Patanarapeelert, Klot Patanarapeelert

**Affiliations:** a Department of Mathematics, Faculty of Applied Science, King Mongkut's University of Technology North Bangkok, Bangkok, 10800, Thailand; b Department of Mathematics, Faculty of Science, Silpakorn Universtiy, Nakhon Pathom Province, 73000, Thailand

**Keywords:** Antibiotic treatment failure, Carrier, Branching process, Parametric extinction threshold

## Abstract

Antibiotic treatment failure related to carriers poses a serious problem to physicians and epidemiologists. Due to the sparsity of data, assessing the role in infection dynamics is difficult. In this study, we examined the possibility that a particular therapeutic effectiveness will be regarded as the disease extinction threshold through the mathematical modelling approach. Including the treatment state in the generic epidemic model with carrier allows us to describe the role of carriers in the treatment failure. The parameterized extinction thresholds were derived via the basic reproduction number for deterministic model, and via the Jury stability criterion for the stochastic model. Existence conditions for the stochastic threshold were derived without the exact formula of the spectral radius of the expectation matrix. The results show that the transmissibility of carrier is necessary for the extinction threshold via treatment failure. The expected extinction threshold may occur subject to the certain range of the transmission potential of the symptomatic infection. This existence conditions are independent of the rate at which the carriers undergo treatment and can be used to support a control strategy.

## Highlights

• Characteristics of antibiotic failure related to carriers were captured through deterministic and stochastic models.
• Parametric extinction thresholds were analyzed for both deterministic and stochastic models.
• Treatment failure can be a parameterized extinction threshold within a certain range of symptomatic transmissibility.

## Graphical abstract

**Figure:**
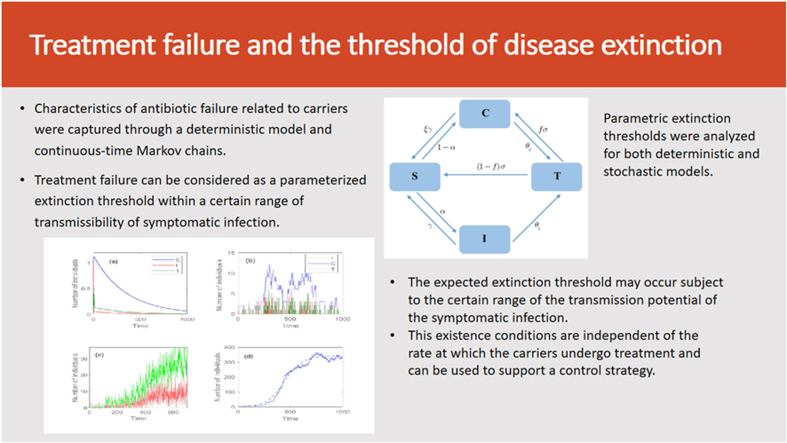

## 1 Introduction

The presence of asymptomatic carriers or *carriers* in many infectious diseases such as Staphylococcus aureus, Streptococcus pneumoniae, enteric fever, Salmonella serovars, influenza and COVID-19 may silently contribute to transmission, subject to an uncertain transmissibility (Chisholm et al., 2018; Gopinath et al., 2012; Gunn et al., 2014; Tan et al., 2022). The term carrier may refer to either pre-symptomatic infections or chronic carriers, with the main difference being the duration of pathogen carriage (Shaikh et al., 2023). The precise role of short-term and chronic carriers in disease transmission remains unclear due to the sparsity of data, even though these asymptomatic carriers presumably act as reservoirs for a diverse range. Although the transmissibility and prevalence of carriers are relatively low with respect to the symptomatic infection, excluding their impact may lead to misleading assessments of pathogen transmission and control.

Theoretically, it is suggested that the transmission potential of symptomatic cases could be overestimated if the relative transmission of carriers is overlooked. This is because the estimated reproduction number should be weighted by the transmission potential of carriers (Anderson & May 1991). Therefore, incorporating carriers into models may reduce uncertainty in estimates of model parameters and improve the evaluation of control strategies.

Practically, the fraction of carriers is unknown during an outbreak. They are unlikely to seek medical care due to the absence of symptoms. However, a secondary difficulty arises when carriers develop symptoms similar to those caused by different pathogens. For instance, the illusive nature of group A *β*-hemolytic streptococcal pharyngitis (GAS) carriers usually embroils a confounding in identification and treatment, leading to the so-call *apparent bacteriologic failure* (Gerber, 1994).

Apart from being symptom-free, it is believed that GAS carrier experience bacteriologic failure following antibiotic therapy that is appropriate for streptococcal pharyngitis (Martin, 2016). A positive result of GAS culture is commonly detected in carriers who had a viral infection, which may lead to unnecessary antimicrobial therapy(Hill, 2010). It is hypothesized that chronic carriers of GAS have no risk for complications from GAS itself or other nonsuppurative (acute rheumatic fever) complications (Speert, 1998). Therefore, avoiding therapy for chronic carriers is suggested to limit the unnecessary use of antimicrobial agents.

Medical research aims to improve prevention measures and interventions, such as early identification of carriers among patients with acute GAS infection, accurate diagnosis of true GAS pharyngitis, and the treatment of carriers. Unfortunately, there is a lack of investigation into the impact of treatment failure on the disease transmission in both theoretical and empirical frameworks. Treatment failure may account for the estimation of carriage rate that is 5–25% (DeMuri & Wald, 2014) and around 6–11% in active sore throat management programmes (Oliver et al., 2018). While the transmissibility of GAS carrier is thought to be ineffective, some studies pointed out that infection from carriers can occur (Pichichero & Casey, 2003). Our study is motivated by these concerns and will examine the impact of bacteriologic failures on the transmission dynamics of group A streptococcus.

A threshold between the extinction and persistence of a disease in a population can be described by the basic reproduction number or *R*_0_, a well-known and widely used quantity in epidemiology. In a model-based approach, *R*_0_ can be estimated from the deterministic compartmental model. In a stochastic framework, the disease extinction threshold is expressed in probabilistic terms (Allen & Lahodny, 2012). The equivalence between these two concepts has been established in a general framework (Allen & van den Driessche, 2013). In addition to potentially improving the assessment of the control strategy, the implication of disease extinction threshold leads to an increased understanding of the underlying mechanism or factors that influence the infection. Given that a set of estimated parameters maps an epidemiological condition to either side of an associated disease extinction threshold, several questions may arise: Can the variation of the treatment failure be responsible for such threshold behavior while the other effects are fixed? If so, under which conditions may the threshold property determined by it occur?

An increase in treatment failure rates with antimicrobial agents, particularly for Streptococcus pyogenes pharyngitis, has been reported. The rate of microbiologic treatment failure for GAS after penicillin treatment has risen from 4-8% to 20–40% over the past 15 years (Pichichero & Casey, 2007). The causes of penicillin failure in eradicating GAS can be broadly classified into three categories: carriers, non-compliance, and various intracellular mechanisms. It was estimated that about half of the patients who harbor GAS following therapy may be carriers (Brook, 2017).

This study aims to investigate whether treatment failure can serve as a disease extinction threshold parameter using mathematical models. We develop a deterministic compartmental model and its associated continuous-time Markov chains, focusing on the carrier state and a parameter that reflects the probability of treatment failure. Consequently, the disease extinction thresholds will be determined through the basic reproduction number and offspring probability-generating functions. Different parameterized thresholds and their existence conditions have been investigated for the deterministic models. For instance, the fraction of potential inter-patch commuters can be the threshold parameter in the gravity model of the movement in patchy meta-population (Patanarapeelert, 2020). The fraction of new infections that develops clinical symptoms was identified as a key measure to determine the outbreak of GAS under the certain conditions (Yokchoo et al., 2019). The extinction threshold and probability of the disease extinction have been studied by applying the multitype Galton-Watson branching process for several stochastic models (Maliyoni, 2021; Zhang et al., 2023). Here, we proposed a slightly distinct approach to determine the parameterized threshold from those studies that might be beneficial to the analysis of higher dimensional system.

## 2 Material and methods

### 2.1 Model assumptions

**Fig. 1:**
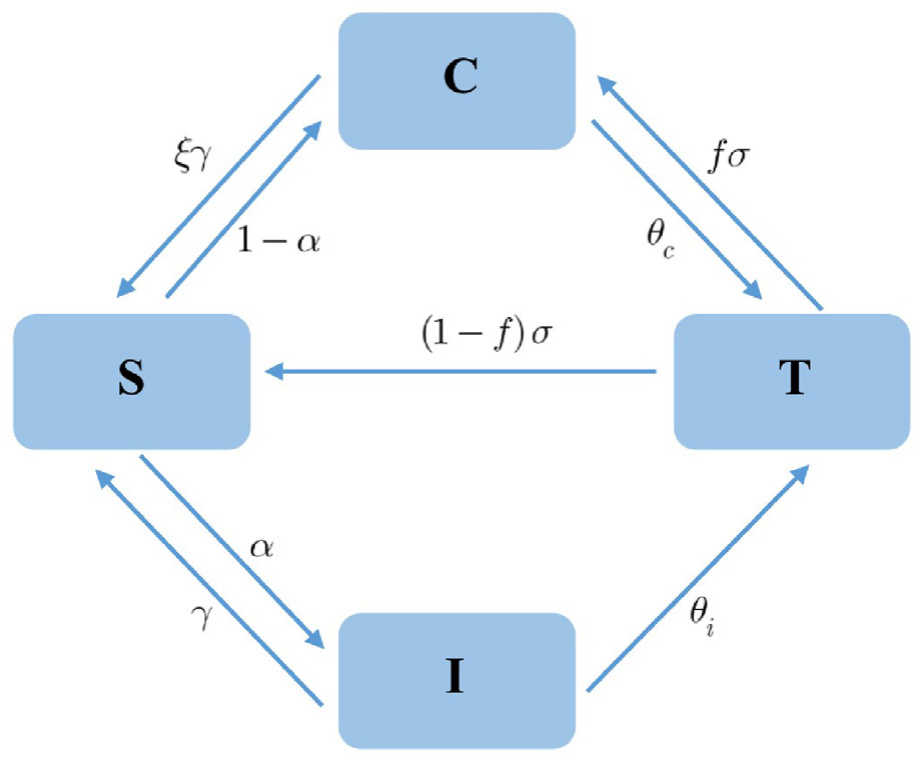
Epidemiological model with carrier and treatment states.

The proposed model was developed based on a previous generic infection dynamics model that incorporates asymptomatic carriers and allows for reinfection (Chisholm et al., 2018). As previously reviewed, the exposed and temporary immune classes are disregarded to keep the model simple as necessary. In this study, the epidemiological states of hosts comprise susceptible (*S*), acute infection with symptoms (*I*), asymptomatic carrier (*C*), and treatment (*T*). An infection cycle shown in Fig. 1 can be described as follows. An infection can cause an individual to become either state *I* with a probability *α* or state *C* with a probability 1 − *α*. Patients in state *T* are mixing of acute and carrier states but commonly in the course of treatment that usually is cautious or may be isolated. Due to little effect in the transmission, we assume that the transmissibility of the treatment class is ineffective for the sake of simplicity. The rates at which carriers and symptomatic individuals undergo treatment are denoted by *θ*_*c*_ and *θ*_*i*_, respectively. We assume that there are no transitions between *C* and *I*. The inclusion of these has been made to emphasize on specific disease and interventions (Chisholm et al., 2018). Treatment failure is captured by the probability *f*. We assume that the treatment normally completed with the rate *σ*. Thus, the success rate is then given by (1 − *f*)*σ*. By the failure, we mean that the eradication of disease pathogens is incomplete. As a result, a patient who fails in therapy by any mean is assumed to move in the carrier state. The self-recovery rate is denoted by *γ*, and *ξ* denotes the scaling factor by which a carrier recovers.

### 2.2 Deterministic and stochastic models

According to the model assumptions, the proposed deterministic compartmental model obeys the following differential equations

(1) $$\begin{array}{lll} \frac{dS}{dt} & = & -\beta \left(I+\eta C\right)S+\sigma \left(1-f\right)T+\gamma I+\gamma \xi C,\\ \frac{dC}{dt} & = & \left(1-\alpha \right)\beta \left(I+\eta C\right)S-\left(\xi \gamma +\theta_{c}\right)C+\sigma fT,\\ \frac{dI}{dt} & = & \alpha \beta \left(I+\eta C\right)S-\left(\gamma +\theta_{i}\right)I,\\ \frac{dT}{dt} & = & \theta_{c}C+\theta_{i}I-\sigma T, \end{array}$$

where the incidence rate obeys the mass action law with the transmission rate *β*. The parameter *η* denotes a scaling factor in the transmissibility of the carrier. It is noted that the total population denoted by *N* is constant.

For the stochastic model, we define a continuous-time Markov chain (CTMC) associated with the deterministic model (1) as

$$\vec{X}\left(t\right)=\left(S\left(t\right),C\left(t\right),I\left(t\right),T\left(t\right)\right),\;t\in \left[0,\infty \right),$$

such that the state space of each component is {0, 1, *…*, *N*}. By the time-homogeneous property, the associated transition probabilities for a small period of time Δ*t* > 0 can be viewed as the probabilities of the change $\Delta \vec{X}\left(t\right)=\vec{X}\left(t+\Delta t\right)-\vec{X}\left(t\right)$ given $\vec{X}\left(t\right)$. This is summarized in Table 1 for all possible events.

**Table 1:** State transitions of the CTMC.

Event	$\Delta \vec{X}\left(t\right)$	Probability
Infection to C	(-1,1,0,0)	(1 − *α*)*λS*Δ*t* + *o*(Δ*t*)
Infection to I	(-1,0,1,0)	*αλS*Δ*t* + *o*(Δ*t*)
Flow from T to S	(1,0,0,-1)	*σ*(1 − *f*)*T*Δ*t* + *o*(Δ*t*)
Flow from T to C	(0,1,0,-1)	*σfT*Δ*t* + *o*(Δ*t*)
Recover from I	(1,0,-1,0)	*γI*Δ*t* + *o*(Δ*t*)
Recover from C	(1,-1,0,0)	*γξC*Δ*t* + *o*(Δ*t*)
Flow from C to T	(0,-1,0,1)	*θ*_*c*_*C*Δ*t* + *o*(Δ*t*)
Flow from I to T	(0,0,-1,1)	*θ*_*i*_*I*Δ*t* + *o*(Δ*t*)

### 2.3 The disease extinction threshold

The basic reproduction number denoted by *R*_0_ is used to estimate the transmission potential of a primary case introduced into a whole naive population. Threshold property implies that if *R*_0_ < 1, then the disease dies out, but if *R*_0_ > 1, the disease spreads. For model (1), *R*_0_ can be derived by the next generation method (van den Driessche & Watmough, 2002) as

(2) $$R_{0}=R_{I}\left(1+\eta \phi \right),$$

where

(3) $$R_{I}=\frac{\alpha \beta N}{\theta_{i}+\gamma },$$

describes the transmission potential of a symptomatic patient in the absence of carriers, and

(4) $$\phi =\frac{\left(1-\alpha \right)\left(\theta_{i}+\gamma \right)+\alpha \theta_{i}f}{\alpha \left(\left(1-f\right)\theta_{c}+\gamma \xi \right)},$$

describes the ratio of carriers over symptomatic infections at endemic state. It vanishes in the absence of carrier and the treatment is assumed to be perfect. It is seen that *R*_*I*_ is superior to *ϕ* in determining the existence of the threshold, i.e., if *R*_*I*_ > 1, then *R*_0_ > 1 for any values of *ϕ* and *η*. In this case, the threshold does not exist. Thus, in order to determine the extinction threshold, one must impose the condition *R*_*I*_ < 1.

In the stochastic model, the probability of disease extinction is defined as

$$P_{\mathrm{ext}}=\underset{t\to \infty }{\lim}Prob\left\{C\left(t\right)=0,I\left(t\right)=0,T\left(t\right)=0\right\}.$$

This probability has a threshold property, i.e., when *P*_*ext*_ = 1, the disease vanishes almost surely, but when *P*_*ext*_ < 1, there is a chance that the disease persists.

We applied the multitype branching process theory to determine the existence condition of the stochastic threshold and to estimate *P*_*ext*_. According to the method, it is necessary to assume that the number of susceptible population is large, an infected individual in each generation is independent to make potential contacts, and the initial number of infected population is small (Allen, 2017; Allen & Lahodny, 2012). Let *f*_*i*_(*u*_1_, *u*_2_, *u*_3_) denote the offspring probability generating function (pgf) for type *i*, where *i* = 1, 2, 3, represent carrier, symptomatic infection, and treatment state, respectively. For instance, *f*_1_(*u*_1_, *u*_2_, *u*_3_) is the offspring pgf for carrier given *C*(0) = 1, *I*(0) = *T*(0) = 0. The probability that an infected individual class *i* causes a random number belonging to class *j* in the next generation, where *i*, *j* = 1, 2, 3 can be calculated using the transition probabilities in Table 1. This gives

(5) $$\begin{array}{lll} f_{1}\left(u_{1},u_{2},u_{3}\right) & = & \frac{\beta N\left(1-\alpha \right)\eta {u_{1}}^{2}+\beta N\alpha \eta u_{1}u_{2}+\theta_{c}u_{3}+\gamma \xi }{\beta N\eta +\gamma \xi +\theta_{c}},\\ f_{2}\left(u_{1},u_{2},u_{3}\right) & = & \frac{\beta N\alpha {u_{2}}^{2}+\beta N\left(1-\alpha \right)u_{1}u_{2}+\theta_{i}u_{3}+\gamma }{\beta N+\gamma +\theta_{i}},\\ f_{3}\left(u_{1},u_{2},u_{3}\right) & = & 1-f+fu_{1}. \end{array}$$

It follows that the expectation matrix is given by

(6) $$M=\left[\begin{array}{lll} \frac{\beta N\eta \left(2-\alpha \right)}{\beta N\eta +\gamma \xi +\theta_{c}} & \frac{\beta N\alpha \eta }{\beta N\eta +\gamma \xi +\theta_{c}} & \frac{\theta_{c}}{\beta N\eta +\gamma \xi +\theta_{c}}\\ \frac{\beta N\left(1-\alpha \right)}{\beta N+\gamma +\theta_{i}} & \frac{\beta N\left(1+\alpha \right)}{\beta N+\gamma +\theta_{i}} & \frac{\theta_{i}}{\beta N+\gamma +\theta_{i}}\\ f & 0 & 0 \end{array}\right].$$

The element of the matrix describes the expected number of offspring of class *i*, produced by a member of class *j*. The spectral radius of *M* denoted by *ρ*(*M*) determines the extinction threshold, i.e., if *ρ*(*M*) < 1, then *P*_*ext*_ = 1, but if *ρ*(*M*) > 1, then *P*_*ext*_ < 1. For latter, there is a unique fixed point of the pgfs, $\bar{u}=\left(\bar{u_{1}},\bar{u_{2}},\bar{u_{3}}\right)\neq 1$, such that

(7) $$P_{\mathrm{ext}}={\bar{u_{1}}}^{C_{0}}{\bar{u_{2}}}^{I_{0}}{\bar{u_{3}}}^{T_{0}},$$

where *C*(0) = *C*_0_,*I*(0) = *I*_0_, and *T*(0) = *T*_0_.

### 2.4 Existence of the parameterized extinction threshold

To see whether a parameter of interest has a threshold property for both deterministic and stochastic models, the conditions for which its critical value exists must be determined. In this study, we mainly focus on the probability of treatment failure *f*. It is hypothesized that the disease is extinct if *f* < *f*_*c*_, and the major outbreak occurs if *f* > *f*_*c*_, for some critical value *f*_*c*_. Although *f*_*c*_ can be calculated, the threshold property might not hold if *f*_*c*_ > 1. In the deterministic model, that critical point *f*_*c*_ is referred to as the value of *f* such that *R*_0_ = 1. In the stochastic model, it is referred to as the value of *f* such that the spectral radius of the expectation matrix is equal to one, i.e., *ρ*(*M*) = 1. Although it has been shown (Allen & van den Driessche, 2013) that the deterministic and stochastic thresholds for disease extinction satisfy the following relationship

(8) $$R_{0}<1\;⟺\;\rho \left(M\right)<1,$$

the equivalence of the existence condition for the extinction threshold that depends on a particular parameter should be ascertained.

The characteristic equation of the expectation matrix (6) is in the form

(9) $$\lambda^{3}+b_{1}\lambda^{2}+b_{2}\lambda +b_{3}=0$$

where *b*_1_, *b*_2_ and *b*_3_ are the functions of parameters. Besides the numerical calculation, it is clear that the closed-form of the roots in this case is impractical. Instead, we employ the side conditions that are necessary and sufficient to have all the roots of a cubic polynomial inside the unit circle in the complex plane (Gardini et al., 2021). These conditions are as follows.

i) 1 + *b*_1_ + *b*_2_ + *b*_3_ > 0,
ii) 1 − *b*_1_ + *b*_2_ − *b*_3_ > 0,
iii) $1-b_{2}-b_{3}^{2}+b_{1}b_{3}>0$,
iv) |*b*_3_| < 1.

Lemma 2.1 *ρ*(*M*) < 1 *if and only if the above four conditions hold*.

### 2.5 The role of carrier and special cases

To understand how carriers takes part in the parameterized threshold, some special cases should be considered based on transmissibility and the rate at which carriers undergo treatment. In most diseases, carrier transmissibility is fairly low due to the absence of symptoms. However, in some diseases such as tuberculosis, hepatitis B virus, and HIV, the ability to transmit can vary among carriers. For Group A Streptococcus, it is possible that some carriers may be able to transmit while others may not (DeMuri & Wald, 2014).

Thus, we aim to address the following questions: First, what happens if the carrier is assumed to be unable to transmit the disease? In the model, this means that *η* = 0. Second, despite carriers being able to transmit the disease, how do they contribute to the threshold if they remain untreated? In the model, this means that *θ*_*c*_ = 0. Analyzing these two special cases may lead to a better understanding about the role of carriers in the general case.

## 3 Results

### 3.1 Analysis of the deterministic model

#### 3.1.1 Special case I

Consider the case *η* = 0, that is the carriers do not transmit the disease. Under this circumstance, the carrier can be viewed as a reservoir which may play a crucial role in the long-term behavior. For the temporal epidemic, it causes the basic reproduction number to reduce to *R*_*I*_ (see Eq. (3)) and is independent of the treatment failure.

#### 3.1.2 Special case II

We now suppose that *θ*_*c*_ = 0. In this case, all carriers are assumed to be untreated, but may be able to transmit the pathogen to others. As a result, the basic reproduction number becomes linearly increasing with *f*, that is

(10) $$R_{0}=a_{0}+a_{1}f,$$

where

(11) $$a_{0}=R_{I}\left(1+\eta \frac{\left(1-\alpha \right)\left(\theta_{i}+\gamma \right)}{\alpha \gamma \xi }\right),\;\;\text{and}\;\;a_{1}=R_{I}\frac{\theta_{i}}{\gamma \xi }.$$

It is seen that if *a*_0_ > 1, then *R*_0_ > 1 for all *f* ∈ [0, 1]. By solving *R*_0_ = 1 for *f*, we obtain

(12) $$f_{c}=\frac{\gamma \xi \left(\theta_{i}+\gamma -\alpha \beta N\right)-\eta \beta N\left(1-\alpha \right)\left(\theta_{i}+\gamma \right)}{\alpha \eta \beta N\theta_{i}}.$$

We thus get the following result.

Theorem 3.1 *Suppose thatθ*_*c*_ = 0 *and let* *a*_0_ *and* *f*_*c*_ *be defined as in* Eqs. (11), (12), *respectively*. *The deterministic extinction threshold via a parameter* *f* *exists if and only if* *a*_0_ < 1 *and* *f*_*c*_ < 1; *i*.*e*., *the disease dies out if* *f* < *f*_*c*_, *and the disease spread if* *f* > *f*_*c*_.

If one defines the transmission potential of carrier without the treatment failure as *R*_*C*_ = (1 − *α*)*βNη*/*γξ*, we are able to write the first condition as

(13) $$a_{0}=R_{I}+R_{C}<1.$$

The first condition in the above theorem tells us that the existence of an extinction threshold for *f* requires that the total transmission potential without the treatment failure must be less than one. It is clear that this condition includes the prior assumption that *R*_*I*_ < 1.

In the second condition, it implies that

(14) $$1-R_{I}<\left(\frac{\beta \eta N}{\gamma \xi }\right)\left(\frac{\theta_{i}+\left(1-\alpha \right)\gamma }{\theta_{i}+\gamma }\right).$$

To satisfy this condition, *R*_*I*_ cannot be too small and is conditioned on the transmission potential of the carrier.

#### 3.1.3 General case

For the deterministic threshold, we first note that *ϕ* in Eq. (4) can be seen as an increasing function of the parameter *f* when the other parameters are held constant. So is *R*_0_, since it is directly proportional to *ϕ*. Let *ϕ*_0_ denotes *ϕ* given *f* = 0, and define the critical value of *f* that generalizes (12) as

(15) $$f_{c}=\frac{\left(\theta_{c}+\gamma \xi \right)\left(\theta_{i}+\gamma -\alpha \beta N\right)-\eta \beta N\left(1-\alpha \right)\left(\theta_{i}+\gamma \right)}{\alpha \eta \beta N\theta_{i}+\theta_{c}\left(\theta_{i}+\gamma -\alpha \beta N\right)}.$$

We obtain the following result.

Theorem 3.2 *Letϕ*_0_*be the value ofϕsuch thatf* = 0, *and* *f*_*c*_ *be defined as in* Eq. (15). *The deterministic extinction threshold via the parameter* *f* *exists if and only if* *R*_*I*_(1 + *ηϕ*_0_) < 1, *and* *f*_*c*_ < 1.

Just as in the previous case, we find that the total transmission potential must be less than one in order to determine the parameterized extinction threshold. In this case, the transmission potential of carrier involves the rate at which a carrier undergoes treatment, i.e., *R*_*C*_ = (1 − *α*)*βNη*/(*θ*_*c*_ + *γξ*). For *f*_*c*_ < 1, we obtain a constraint for *R*_*I*_ in the same manner as in the previous case.

### 3.2 Analysis of the stochastic model

#### 3.2.1 Special case I

In the case *η* = 0, the expectation matrix (6) becomes

(16) $$M=\left[\begin{array}{lll} 0 & 0 & \frac{\theta_{c}}{\gamma \xi +\theta_{c}}\\ \frac{\beta N\left(1-\alpha \right)}{\beta N+\gamma +\theta_{i}} & \frac{\beta N\left(1+\alpha \right)}{\beta N+\gamma +\theta_{i}} & \frac{\theta_{i}}{\beta N+\gamma +\theta_{i}}\\ f & 0 & 0 \end{array}\right].$$

The corresponding characteristic equation reads

(17) $$\left(\lambda^{2}-\frac{\theta_{c}f}{\gamma \xi +\theta_{c}}\right)\left(\lambda -\frac{\beta N\left(1+\alpha \right)}{\beta N+\gamma +\theta_{i}}\right)=0.$$

It is easy to see that the spectral radius of *M* in this case is uniquely determined by the root of the second term of the above equation, since the absolute values of the roots of the first term are always less than one. Treatment failure has nothing to do with the stochastic extinction threshold in this case. It is also observed that

$$\frac{\beta N\left(1+\alpha \right)}{\beta N+\gamma +\theta_{i}}<1\;⟺\;R_{0}<1.$$

To estimate *P*_*ext*_, we solve for the fixed point of system (5) under the constraint *R*_0_ > 1. After some calculations, we have ${\bar{u}}_{1}={\bar{u}}_{3}=1$ and ${\bar{u}}_{2}=1/R_{0}$. We then have the following results.

Theorem 3.3 *In the case thatη* = 0, *the disease is extinct with probability one if* *R*_0_ < 1. *On the other hand*, *it is extinct with probability*,

(18) $$P_{\mathrm{ext}}={\left(\frac{1}{R_{0}}\right)}^{I_{0}},$$

*if* *R*_0_ > 1.

#### 3.2.2 Special case II

By substituting *θ*_*c*_ = 0 into Eq. (5), we obtain

(19) $$M=\left[\begin{array}{lll} \frac{\beta N\eta \left(2-\alpha \right)}{\beta N\eta +\gamma \xi } & \frac{\beta N\alpha \eta }{\beta N\eta +\gamma \xi } & 0\\ \frac{\beta N\left(1-\alpha \right)}{\beta N+\gamma +\theta_{i}} & \frac{\beta N\left(1+\alpha \right)}{\beta N+\gamma +\theta_{i}} & \frac{\theta_{i}}{\beta N+\gamma +\theta_{i}}\\ f & 0 & 0 \end{array}\right].$$

We first note that if *f* = 0, then the above matrix can be simplified so that the necessary condition for the existence of the threshold can be derived. To this end, we denote *K* as a left upper 2 × 2 sub-matrix of *M*. It is easy to see that if *f* = 0, then the nonzero eigenvalues of *M* are determined by the characteristic equation

(20) $$\lambda^{2}-tr\left(K\right)\lambda +\det\left(K\right)=0,$$

where

$$tr\left(K\right)=\frac{\beta N\eta \left(2-\alpha \right)}{\beta N\eta +\gamma \xi }+\frac{\beta N\left(1+\alpha \right)}{\beta N+\gamma +\theta_{i}},$$

and

$$\det\left(K\right)=\frac{2{\left(\beta N\right)}^{2}\eta }{\left(\beta N\eta +\gamma \xi \right)\left(\beta N+\gamma +\theta_{i}\right)}.$$

According to the Jury condition, we subsequently see that *ρ*(*K*) < 1 iff

(21) tr(K)<1+det(K)<2.

Thus, in the case that *θ*_*c*_ = 0 and *f* = 0, the disease is extinct almost surely if and only if the above conditions are satisfied. Likewise, we observed that the first inequality tr(*K*) < 1 + det(*K*) is equivalent to the condition *a*_0_ < 1 in the deterministic case.

We now consider the case *f* > 0. To bring about the threshold value of *f*, condition (21) must be met. Otherwise, there is no chance for the disease extinction with probability one since the more treatment failure there are, the more accumulated carriers foster the infection by assumption. The characteristic equation of *M* when *f* ≥ 0 is given by

(22) $$\lambda^{3}-tr\left(K\right)\lambda^{2}+\det\left(K\right)\lambda -f\Delta =0,$$

where an additional parameter is defined by

(23) $$\Delta =\left(\frac{\beta N\alpha \eta }{\beta N\eta +\gamma \xi }\right)\left(\frac{\theta_{i}}{\beta N+\gamma +\theta_{i}}\right).$$

We now examine conditions such that the parametric threshold exists by employing Lemma 2.1. To satisfy the condition (i), we must have

(24) $$f<\frac{1-tr\left(K\right)+\det\left(K\right)}{\Delta }.$$

By substitution, we observe that the right hand side of the above inequality is identical to the expression of *f*_*c*_, the threshold value of *f* in the deterministic model. Therefore, we must show that the remaining conditions (ii)-(iv) in Lemma 2.1 give the same result as the condition (i) or are satisfied for any value of *f* ∈ [0, 1]. In the latter, it implies that they are not useful for determining the threshold value.

Since the condition (ii) gives

(25) 1+tr(K)+det(K)+Δf>0,

it is obvious that threshold does not exist.

In condition (iii), we get

(26) $$\Delta^{2}f^{2}-\Delta tr\left(K\right)f+\det\left(K\right)-1>0.$$

By Jury condition (21), it follows that

(27) $$0<f<\frac{tr\left(K\right)+\sqrt{tr{\left(K\right)}^{2}+4\left(1-\det\left(K\right)\right)}}{2\Delta }.$$

Since

(28) $$\frac{tr\left(K\right)}{\Delta }=\frac{1}{\alpha }\left[1+\frac{\eta \left(2-\alpha \right)\left(\beta N+\gamma \right)+\left(1-\alpha \right)\eta \theta_{i}+\left(1+\alpha \right)\left(\beta N\eta +\gamma \xi \right)}{\eta \theta_{i}}\right]>1,$$

the condition (iii) in Lemma 2.1 holds for all *f* ∈ [0, 1].

The last condition in lemma 2.1 is given by

(29) $$f<\frac{1}{\Delta }.$$

Since

(30) $$\frac{1}{\Delta }=\left[\frac{1}{\alpha }+\frac{\gamma \xi }{\beta N\alpha \eta }\right]\left[1+\frac{\beta N+\gamma }{\theta_{i}}\right]>1,$$

the condition holds for all *f* ∈ [0, 1]. Thus, the existence conditions for the stochastic threshold can be summarized in the following theorem.

Theorem 3.4 *Suppose thatθ*_*c*_ = 0 *and let* *f*_*c*_ *be defined as in* Eq. (12). *A stochastic extinction threshold via* *f* *exists if and only if the Jury condition (21) holds and* *f*_*c*_ < 1. *The threshold value*, *if it exists*, *is the same as in the deterministic case*, *i*.*e*., *the disease is extinct with probability one if* *f* < *f*_*c*_, *or is extinct with probability less than one if* *f* > *f*_*c*_.

The fixed point of the offspring pgfs in the case *f* > *f*_*c*_ can be derived by solving for *u*_*i*_, *i* = 1, 2, 3 of the system

(31) $$\begin{array}{lll} u_{1} & = & \frac{\beta N\left(1-\alpha \right)\eta {u_{1}}^{2}+\beta N\alpha \eta u_{1}u_{2}+\gamma \xi }{\beta N\eta +\gamma \xi }, \end{array}$$

(32) $$\begin{array}{lll} u_{2} & = & \frac{\beta N\left(1-\alpha \right)u_{1}u_{2}+\beta N\alpha {u_{2}}^{2}+\theta_{i}u_{3}+\gamma }{\beta N+\gamma +\theta_{i}}, \end{array}$$

(33) $$\begin{array}{lll} u_{3} & = & 1-f+fu_{1}. \end{array}$$

#### 3.2.3 General case

To establish the existence condition in the stochastic model, we consider *K*, a sub-matrix of the expectation matrix *M* in (6) given *f* = 0. It is noticed that the matrix *K* in this case is different from the previous case. As in the previous case, the Jury condition must be held to insure the existence of the threshold where

(34) $$tr\left(K\right)=\frac{\beta N\eta \left(2-\alpha \right)}{\beta N\eta +\gamma \xi +\theta_{c}}+\frac{\beta N\left(1+\alpha \right)}{\beta N+\gamma +\theta_{i}},$$

and

(35) $$\det\left(K\right)=\frac{2{\left(\beta N\right)}^{2}\eta }{\left(\beta N\eta +\gamma \xi +\theta_{c}\right)\;\left(\beta N+\gamma +\theta_{i}\right)}.$$

We have a characteristic equation of *M* as

(36) $$\lambda^{3}-tr\left(K\right)\lambda^{2}+\left(\det\left(K\right)-f\omega \right)\lambda -f\Delta =0,$$

where tr(*K*), det(*K*) are given as above, and additional parameters are given by

(37) $$\omega =\frac{\theta_{c}}{\beta N\eta +\gamma \xi +\theta_{c}},$$

and

(38) $$\Delta =\frac{\beta N\alpha \eta \theta_{i}-\beta N\left(1+\alpha \right)\theta_{c}}{\left(\beta N\eta +\gamma \xi +\theta_{c}\right)\left(\beta N+\gamma +\theta_{i}\right)}.$$

It is worth noting that the value of the parameter Δ is not always positive as in the special case II.

The first condition in Lemma 2.1 gives

(39) $$f<\frac{1-tr\left(K\right)+\det\left(K\right)}{\omega +\Delta }.$$

Since

$$\omega +\Delta =\frac{\beta N\alpha \eta \theta_{i}+\theta_{c}\left(\theta_{i}+\gamma \right)\left(1-R_{I}\right)}{\left(\beta N\eta +\gamma \xi +\theta_{c}\right)\left(\beta N+\gamma +\theta_{i}\right)}>0,$$

and due to the Jury condition, we see that the right hand side term of condition (39) is positive. Again, it can be shown that the right hand side term in (39) is indeed the critical value *f*_*c*_ in Eq. (15). By following the proof in the previous case, it is easy to see that the remaining conditions hold through the range of *f*, if Δ ≥ 0. However, the conclusion might not be true for Δ < 0. To cope with this problem we add an another condition by supposing that $|\Delta |<\sqrt{1-\det\left(K\right)}$. It is immediate that the last condition in Lemma 2.1 holds. Thus, the remaining task is to show that the second and the third conditions are true for all *f*.

To satisfy the second condition in Lemma 2.1, we must have

1+tr(K)+det(K)−fω+fΔ>0.

By some inspection, it is seen that tr(*K*) + *f*Δ > 0. Since 1 − *fω* > 0, the above condition holds for all *f* ∈ [0, 1].

After substitution, the condition (iii) can be rearranged as

(40) $$\Delta^{2}f^{2}-\left(\omega +\Delta tr\left(K\right)\right)f-\left(1-\det\left(K\right)\right)<0.$$

The left expression can be viewed as a quadratic polynomial of *f*. Since det(*K*) < 1, the condition holds for *f*_−_ < *f* < *f*_+_, where *f*_−_ and *f*_+_ are two real roots of the polynomial. It is obvious that *f*_−_ < 0 and *f*_+_ > 0. We now consider the sign of the polynomial at *f* = 1, i.e.,

$$\Delta^{2}-\left(tr\left(K\right)\Delta +\omega \right)-\left(1-\det\left(K\right)\right).$$

Since tr(*K*)Δ + *ω* > 0, and by the additional condition, we have

$$\Delta^{2}-\left(tr\left(K\right)\Delta +\omega \right)-\left(1-\det\left(K\right)\right)<\Delta^{2}-\left(1-\det\left(K\right)\right)<0.$$

This implies that *f*_+_ > 1. Hence, the condition (iii) holds for all *f* ∈ [0, 1]. The results are summarized in the following theorem.

Theorem 3.5 *Suppose thatf*_*c*_*is defined as in* Eq. (15). *A stochastic extinction threshold via* *f* *exists if*

(i) *the Jury condition for a sub-matrix of the expectation matrix (6) holds*,
(ii) *f*_*c*_ < 1, *and*
(iii) $|\Delta |<\sqrt{1-\det\left(K\right)}$.

The threshold value, if it exists, is the same as in the deterministic case, i.e., the disease is extinct with probability one if *f* < *f*_*c*_, or is extinct with probability less than one if *f* > *f*_*c*_.

### 3.3 Numerical simulation

The purpose of numerical calculation is to test and refine the theoretical predictions. To this end, the values of parameters must be specified. Here, the selection of parameter values is based on the epidemiological modeling of Group A streptococcus (GAS) (Yokchoo et al., 2019).

The total population size is set at one thousand individuals. The transmission rate of symptomatic GAS infection is likely the most sensitive parameter for determining the basic reproduction number. We find that using a specific value from previous work tends to violate the condition for the existence of the extinction threshold that is *R*_*I*_ > 1. We thus reduce its magnitude to *β* = 0.0005. Accordingly, the fraction of the carrier transmission rate over the symptomatic transmission rate is adjusted to *η* = 0.1. The value of *α* can vary across the population. For instance, a longitudinal study over a two-year period estimated the carrier rate of beta-hemolytic streptococci at 19.4%, with the GAS symptomatic rate around 5.0% (Strömberg et al., 1988). Another prospective study indicates that the incidence of GAS carriers among healthy children was 2.5%. Therefore, the estimated value of *α* should be as high as 80–90%. We select a high symptomatic incidence rate, with *α* set to 0.8, to offset the impact of carrier transmission. The recovery rate of GAS infection conditioned on the perfect treatment is between 0.1 to one per day. We choose *γ* = 0.5 and *σ* = 0.4.

The rate at which symptomatic cases undergo treatment is not currently found in the literature. Here, we assume that, on average, 75% of symptomatic cases undergo treatment per day, so *θ*_*i*_ = 0.75. In the context of GAS carriers, the value of *θ*_*c*_ should be smaller than *θ*_*i*_. However, estimates of *θ*_*c*_ are rare; for example, one study found a 6.9% treatment rate among children with upper respiratory tract infections, including sore throat, from whom viruses were identified (Pichichero et al., 1999).

To achieve the existence condition, we assume that, on average, 1.2% of carriers seek medical care per day, so *θ*_*c*_ = 0.012. Finally, the fraction of carrier's recovery rate relative to that of symptomatic case is set to *ξ* = 0.016. This implies that the carrier period is assumed to be 62.5 times longer than the infectious period of a symptomatic case. In this example, the average carrier period is about four months. This value is close to the midpoint of the estimated GAS carrier period (3–34 weeks) obtained from observation (Martin et al., 2004).

By using these parameter values, we calculated the condition in Theorem 3.2 as *R*_*I*_(1 + *ηϕ*_0_) = 0.82, so that the critical value of the treatment failure is *f*_*c*_ = 0.11. As the Jury condition is equivalent to such conditions, we only computed Δ = 0.1567, and $\sqrt{1-\det\left(K\right)}=0.7694$. Therefore, the conditions in Theorem 3.5 are satisfied.

**Fig. 2:**
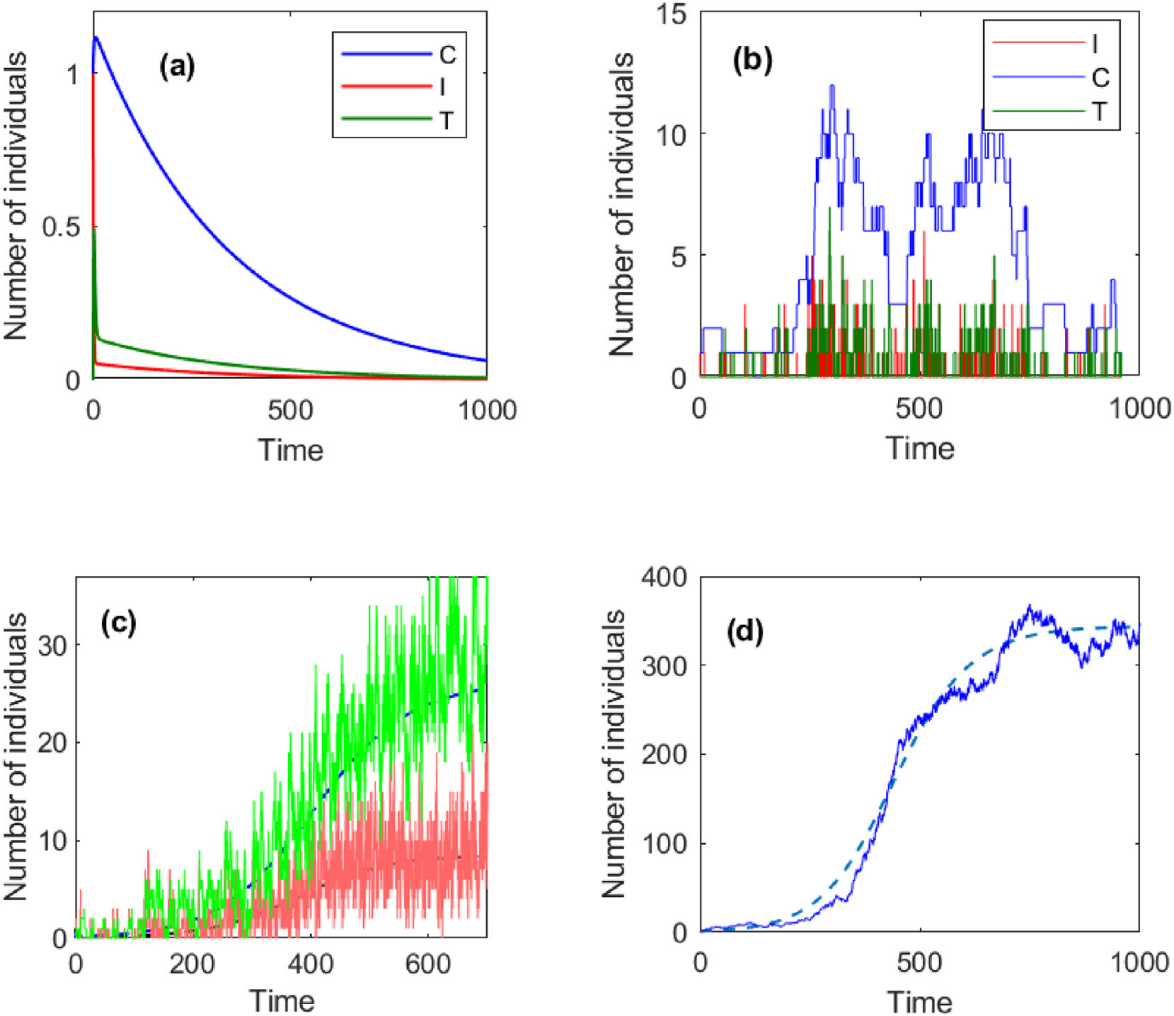
Numerical simulations of the trajectories over time in general case. (a) Solutions of the deterministic model and (b) sample paths of the stochastic model those characterize the disease extinction. (c) Solution of the deterministic model versus sample paths for treatment (green) and symptomatic infection (red) those characterize the major outbreak. (d) Solution of the deterministic model versus a sample path for carrier as in (c).

In the case of *f* = 0.05, the solution to the deterministic model (1) and a sample path of the associated CTMC model are demonstrated in Fig. 2(a) and (b), respectively. Gillespie algorithm (Allen, 2017) was applied to the stochastic simulation. The results show that the extinction of the disease (GAS) happens in each particular sense as predicted by theory. In the case of *f* = 0.4, we computed a fixed point *u*_1_ = 0.57, *u*_2_ = 0.82, and *u*_3_ = 0.82 of the offspring pgfs. By taking *C*_0_ = *I*_0_ = 1, we can calculate the probability of extinction, i.e., *P*_*ext*_ = 0.47. The deterministic curves and a sample path are demonstrated in Fig. 2(c) and (d). The results show that the number of infected individuals (all types) continually increases, where the increase of the number of carriers is relatively high (Fig. 2 (d)). The reason behind this is that the transmission potential of carrier is *R*_*C*_ = 0.5 which is greater than of symptomatic case *R*_*I*_ = 0.32.

In Table 2, we calculated the probability of extinction with respect to the distribution of initial conditions. The result from numerical simulations align well with those directly calculated from the equations.

**Table 2:** Probability of extinctions calculating from the solutions of the fixed point equations and simulation with respect to the distribution of the initial conditions of infected states.

(*C*_0_, *I*_0_, *T*_0_)	direct *P*_*ext*_	approx. *P*_*ext*_
(1,0,0)	0.5715	0.5724
(0,1,0)	0.8222	0.8198
(0,0,1)	0.8286	0.8296
(2,0,0)	0.3266	0.3198
(0,2,0)	0.6760	0.6750
(0,0,2)	0.6866	0.6872
(1,1,0)	0.4700	0.4808
(1,0,1)	0.4735	0.4838
(0,1,1)	0.6812	0.6788

As the assumption of Branching process approximation holds, we computed only for the small initial number. It is clear that the higher the initial number of infected individuals, the lower the probability of extinction. Since there is a chance of complete disease eradication, the probability of extinction when initialized by an individual in the treatment state is somewhat higher than when initialized with a carrier. In this example, the probability of extinction when initialized with the treatment state is similar to the probability when initialized with a symptomatic case. We observe that the resulting pattern is driven by the transmission potential of each infectious state.

## 4 Discussion

The existence of a parameterized extinction threshold highlights a key parameter for supporting a control strategy. In this case, the effectiveness of treatment is focused since carriers are able to transmit the disease. Even if the transmission potential of the carrier is low, reducing the contact rate alone may not be sufficient to achieve the goal of reducing the basic reproduction number to less than one. If we combine condition (13) with condition (14) in general case, we obtain

(41) $$\frac{1}{1+\left(\eta /\alpha \gamma \xi \right)\left(\theta_{i}+\left(1-\alpha \right)\gamma \right)}<R_{I}<\frac{1}{1+\left(\eta /\alpha \gamma \xi \right)\left(1-\alpha \right)\left(\theta_{i}+\gamma \right)}.$$

Once the contact rate is contained, such that the above condition is satisfied, the effectiveness of treatment must be higher than 1 − *f*_*c*_ to ensure that if the epidemic occurs, it will be minor. Otherwise, one must contain the contact rate such that *R*_*I*_ less than the left hand side of the above condition. Evaluation and comparison of control efforts between these two scenarios is a public health challenge.

It is interesting that the existence condition is independent of *θ*_*c*_. One technical reason is that the model does not assume a relationship between *θ*_*c*_ and *f*. A possible underlying hypothesis is that carriers may create confusion in evaluating patients with symptoms similar to those of acute infection. In the context of GAS infection, bacteriologic failure may occur when a GAS carrier with a viral illness has throat cultures positive for S. pyogenes. With viral symptoms, such a patient is less likely to experience a rapid improvement with antibiotic therapy.

However, the model assumption does not imply only treatment failure solely due to confusion in evaluation, as the treatment group includes both carriers and individuals with acute infections. For streptococcal infection, differentiating between the two states is not practical in a clinical setting (DeMuri & Wald, 2014). Nevertheless, it is possible to see how *f*_*c*_ depends on *θ*_*c*_. This can be done by differentiating *f*_*c*_ with respect to *θ*_*c*_. The result indicates that if the existence condition for the extinction threshold holds, then *f*_*c*_ increases with *θ*_*c*_, and vice versa. The explanation is simple: if the effectiveness of treatment is important for disease extinction, increasing the outflow rate of carriers reduces the transmission potential of this group, thereby increasing the chance of disease extinction.

In the stochastic threshold analysis, our method has a technical advantage in determining the condition for a parameterized threshold without needing the exact formula for the spectral radius of the expectation matrix. This implies that once the transition probabilities are defined, the parametric threshold condition can be analyzed without relying on the basic reproduction number. The present method is suitable for problems involving the determination of the parameterized extinction threshold and the stability analysis of the discrete-time dynamical systems subject to the third-order polynomial characteristic equation.

## 5 Conclusions

We showed a possible link between treatment failure and the disease extinction threshold through the role of carriers. The epidemic model was developed for general infectious diseases based on the hypothesis that an infected individual can become a carrier through infection and experiences treatment failure. Further modifications to the model depend on the specific purpose, disease characteristics, available data, and host behavior. For instance, to investigate the impact of vaccination, the immune response compartment may be added. If the disease progression from carrier to acute infection is possible, then the transition from *C* to *I* should be included.

The basic reproduction number can be decomposed into the transmissibility of the symptomatic cases and of the carriers. Unlike the basic reproduction number, treatment failure is responsible for determining a disease extinction threshold under certain conditions. The existence conditions derived from the deterministic model and the stochastic models differ in form but implicitly equivalent.

In deterministic approach, two conditions are derived directly from the basic reproduction number: one by setting the basic reproduction number to be less than one under the perfect treatment, and the other by calculating the critical value of treatment failure and ensuring it is less than one. In the stochastic approach, the existence conditions are given by the Jury stability criterion. The first condition is derived assuming perfect treatment, while the second condition is the same as in the deterministic model.

Although more complex, the derivation of existence conditions in the stochastic model does not utilize the formula of the basic reproduction number or the spectral radius of the expectation matrix. Therefore, the parameterized extinction threshold in the stochastic model can be determined by analyzing the Jury stability criterion. The equivalence of deterministic and stochastic approach ensures that the derivation of conditions in the stochastic model requires only the construction of transition probabilities by any means.

We have found that the carrier state plays an important role in threshold behavior, unless carriers are not allowed to transmit the disease (see the first special case). In addition to the discussed control strategy, which involves evaluating treatment efficacy, the development of effective diagnostic methods to distinguish carriers from acute infections may be necessary.

However, our understanding of carriers is still limited. For instance, questions such as whether and how the transmissibility of carriers in certain diseases wanes over time, and whether they can be reinfected or infected with different stains, are areas for future investigations that could alter the threshold behavior.

## CRediT authorship contribution statement

**Pichaya Voottipruex:** Writing – review & editing, Visualization, Validation, Software, Investigation. **Nichaphat Patanarapeelert:** Writing – review & editing, Validation, Investigation, Formal analysis, Conceptualization. **Klot Patanarapeelert:** Writing – review & editing, Writing – original draft, Validation, Methodology, Investigation, Formal analysis, Conceptualization.

## Declaration of competing interest

The authors declare that they have no known competing financial interests or personal relationships that could have appeared to influence the work reported in this paper.
